# Microbiological Profiles of Dental Implants in Metabolic Syndrome Patients: A Case-Control Study

**DOI:** 10.3390/antibiotics10040452

**Published:** 2021-04-16

**Authors:** Bianca Di Murro, Marta Moretti, Enrico De Smaele, Claudio Letizia, Carla Lubrano, Pier Carmine Passarelli, Antonio D’Addona, Giorgio Pompa, Piero Papi

**Affiliations:** 1 Department of Oral and Maxillo-Facial Sciences, “Sapienza” University of Rome, 00161 Rome, Italy; bianca.dimurro@uniroma1.it (B.D.M.); giorgio.pompa@uniroma1.it (G.P.); 2 Department of Experimental Medicine, Sapienza University of Rome, 00161 Rome, Italy; marta.moretti@uniroma1.it (M.M.); enrico.desmaele@uniroma1.it (E.D.S.); 3 Unit of Secondary Arterial Hypertension, Department of Translational and Precision Medicine, “Sapienza” University of Rome, 00161 Rome, Italy; claudio.letizia@uniroma1.it; 4 Section of Medical Pathophysiology, Food Science and Endocrinology, Department of Experimental Medicine, Sapienza University of Rome, 00161 Rome, Italy; carla.lubrano@uniroma1.it; 5 Oral Surgery and Implantology Unit, Department of Head and Neck, Institute of Clinical Dentistry, Catholic University of Sacred Hearth, Fondazione Policlinico Universitario Gemelli, 00168 Rome, Italy; piercarminepassarelli@hotmail.it (P.C.P.); antonio.daddona@unicatt.it (A.D.)

**Keywords:** dental implants, peri-implantitis, metabolic syndrome, real-time PCR, microbiologic contamination

## Abstract

There is a lack of knowledge on the possible influence of systemic conditions on peri-implantitis. The aim of this case-control study is to evaluate the difference in terms of oral pathogens’ concentrations in the peri-implant sulcus of a group of patients affected by metabolic syndrome (Mets) compared to healthy subjects. For each patient, peri-implant sulcular biofilm samples were obtained by inserting two sterile endodontic paper points in the deepest aspect of the peri-implant sulcus for 30 s. The quantitative real-time polymerase chain reaction was performed to evaluate total bacterial counts of six pathogens. Patients were screened for peri-implant diseases and clinical and radiographic parameters were recorded. A total of 50 patients was enrolled in the study, 25 affected by Mets and 25 healthy. Significantly higher bacterial counts were discovered for *Aggregatibacter actinomycetemcomitans* (*p* = 0.0008), *Prevotella intermedia* (*p* = 0.0477) and *Staphylococcus aureus* (*p* = 0.034) in MetS patients compared to healthy subjects. Performing a sub-group analysis, considering peri-implant status and dividing patients by MetS diagnosis, no statistically significant (*p* < 0.05) differences were found. For the first time, a correlation between MetS presence and a greater prevalence of some bacterial species in the peri-implant sulcus was reported, irrespectively from peri-implant status (health vs. disease).

## 1. Introduction

Implant-prosthetic rehabilitations have demonstrated long-term survival rates (> 10 years), however, the massive use of dental implants over the last decades has also led to the development of “peri-implant diseases”, represented by mucositis and peri-implantitis [1,2,3,4].

According to the 2017 World Workshop on Periodontal and Peri-Implant Diseases and Conditions, mucositis is defined by the presence of bleeding and/or suppuration on gentle probing without loss of supportive bone detected radiographically and peri-implantitis is characterized by the same signs of inflammation with bone loss [5]. Peri-implantitis is a chronic inflammatory disease, considered sensitive to factors inducing tissue inflammation and systemic oxidative stress [6]. Nevertheless, only few papers have studied the relationship between systemic conditions and peri-implant diseases [7,8,9,10,11]. Contradictory results have been reported on the possible relationship between cardiovascular diseases (CVDs) and peri-implantitis [7,9], therefore there is not enough evidence to draw clear conclusions [10,11]. On the contrary, a meta-analysis by Monje et al. [8] reported a 50% higher risk of detecting peri-implantitis in subjects with diabetes/hyperglycaemia compared to non-diabetes patients. Our study group has recently highlighted for the first time that patients affected by Metabolic Syndrome (MetS) showed a strong association with peri-implant mucositis (Odds Ratio = 10.01, *p* = 0.005) and even stronger with peri-implantitis (Odds Ratio = 15.26, *p* = 0.001) [12]. MetS represents a cluster of conditions associated with an increased risk of developing CVDs and type II diabetes [13], with an overall prevalence greater than 40% among adults in US and Europe [14]. It includes at least three of the following parameters: arterial hypertension (HT), hyperglycemia, hypertriglyceridemia, low levels of high-density lipoprotein (HDL) cholesterol and abdominal obesity [13]. The up-regulation of pro-inflammatory cytokines (e.g., IL-1, IL-6, IL-8, and TNF-alpha) has been also hypothesized as a possible cause of reduced insulin sensitivity and endothelial disfunction, important factors in MetS and CVDs development [15,16]. Peri-implant diseases are plaque-related inflammatory conditions and peri-implant mucosal inflammation could represent a trigger for systemic inflammation [17].

Among other risk factors, also the lack of an appropriate band of keratinized mucosa around dental implants might contribute to the development of peri-implant diseases [18,19,20].

Hence, the peri-implant sulcus presents a conformation more vulnerable to pathogens infection [17]: the gingival tissue around the implant neck shows a deeper sulcus that can carry fluids and bacteria up to the implant-abutment junction, creating a deposit for oral pathogens [21,22], with an extension of the inflammatory cell infiltrate more apical than in teeth affected by periodontitis [23]. Periodontitis and peri-implantitis share several clinical features and etiological factors, with some Gram-negative anaerobe bacteria (*Porphyromonas gingivalis, Prevotella intermedia* and *Aggregatibacter actinomycetemcomitans*) strongly associated with the two conditions [23,24].

A recent systematic review [25] compared the microbiological profiles of periodontitis and peri-implantitis: based on their results, even healthy implants are colonized by periodontopathic bacteria, with *P. gingivalis*, *P. intermedia* and *P. nigrescens* being more prevalent in implants affected by peri-implantitis. Compared with periodontitis, peri-implantitis microbiological profile is more heterogeneous and complex, with presence also of *S. aureus* and other anaerobic Gram-positive rod associated species.

Several studies have investigated the microbiological profile of patients affected by peri-implantitis [26,27,28,29], however there is currently a lack of knowledge on the possible influence of systemic conditions on peri-implant sulcus composition. Understanding bacterial and inflammatory activity may help us to target appropriate antibiotics for every patient, contributing to increment the low success rate of currently adopted peri-implantitis treatment strategies. Furthermore, exploring the relationship between metabolic syndrome and peri-implant diseases could lead to a more conscious approach while treating these patients.

Therefore, the aim of this case-control study is to evaluate the difference in terms of oral pathogens’ concentrations in the peri-implant sulcus of a group of patients affected by MetS compared to healthy subjects.

## 2. Results

### 2.1. Implant Data

Subjects enrolled had total of 156 dental implants placed, with a mean of 3.12 implants per patient. The mean functional time was 6.47 ± 4.56 years (range: 5–14 years). At patient-level, prevalence of mucositis was 42%, of peri-implantitis of 26% and of healthy-implants of 32%. At implant-level, 20.51% of dental implants was affected by peri-implantitis, 46.79% by mucositis and 32.70% were healthy implants. Mean PPD values were 3.23 mm ± 1.15, mean MBL levels were 1.27 ± 1.12 mm. Detailed characteristics of study implants are reported in Table 1.

When analyzing peri-implant parameters considering metabolic syndrome diagnosis, only BOP and PI showed statistically significant higher values (*p* < 0.05). No statistically significant differences (*p* > 0.05) were detected for peri-implant diseases prevalence among the two groups.

### 2.2. Microbiological Analysis

Evaluating microbiological samples based on peri-implant status (healthy vs. disease), statistically significant higher total bacterial counts were found out for *P. intermedia* (Pi) (*p* = 0.0332), *P. gingivalis* (Pg) (*p* < 0.001), *F. nucleatum* (Fn) (*p* < 0.001) and *T. Denticola* (Td) (*p* < 0.001) in patients with implants affected by peri-implant diseases (Table 2).

Interestingly, irrespective from peri-implant status, statistically significant higher total bacterial counts were discovered for *A. actinomycetemcomitans* (*p* = 0.0008), *P. intermedia* (*p* = 0.0477) and *S.s Aureus* (*p* = 0.034) in metabolic syndrome patients compared to healthy subjects (Table 3).

When performing a sub-group analysis, considering peri-implant status and dividing patients by metabolic syndrome diagnosis, no statistically significant (*p* > 0.05) differences were found.

## 3. Discussion

The aim of this case-control study was to evaluate the difference in terms of oral pathogens’ concentrations in the peri-implant sulcus of a group of patients affected by MetS compared to healthy subjects. To the best of the authors’ knowledge, this is the first article reporting data on the influence of MetS on the composition of the microbial flora of the peri-implant sulcus.

Since there are no studies published investigating microbiological profiles of dental implants in metabolic syndrome patients, sample size calculation was not possible and we had to enroll an arbitrary number of patients.

Based on our results, statistically significant higher total bacterial counts were found out for *A. actinomycetemcomitans*, *P. intermedia* and *S. aureus* in metabolic syndrome patients compared to healthy subjects, irrespectively from peri-implant status. Therefore, our data suggest a greater prevalence of these oral pathogens in MetS subjects. The literature is controversial on this topic and there are no systematic reviews to which our findings can be compared. Recently, a survey on a large adult population in the US [30] reported that MetS was not associated with a greater prevalence of periodontal bacteria. Only a moderate association with periodontal bacterial profile was found for elevated fasting plasma glucose values, while the other MetS components showed no statistically significant relationship.

On the contrary, Iwasaki et al. [31] found that Japanese MetS patients were 2.9 times more likely to have elevated serum antibody to *P. gingivalis*, while Thanakun et al. [32] reported an association between a lower IgG antibody response to *A*. *actinomycetemcomitans*, but not *P. gingivalis,* and MetS. Hyvärinen et al. [33] confirmed the association of systemic exposure to *A*. *actinomycetemcomitans,* and not with P. gingivalis, reporting an OR of 1.42 for MetS patients.

In a cross-sectional study, Lachmann et al. [34] reported for the first time that CVD was statistically significantly associated with *P. intermedia* prevalence in the peri-implant sulcus. In recent years, several authors have linked *Pi* and other periodontal pathogens with CVDs and the latest joint workshop of the EFP/ WHF [35] confirmed the available evidence on the presence of periodontal pathogens (*P. gingivalis, P. intermedia, F. nucleatum, T. Denticola*) in atheroma lesions. However, while the association between periodontitis and CVDs is well known, the relationship between CVDs and peri-implant diseases is still controversial and there is not enough evidence to draw clear conclusions [10].

Main limitations of our study are represented by its case-control design, with the impossibility to draw clear cause-effect conclusions, and the small sample enrolled.

Furthermore, only a few pathogens were investigated by means of real-time PCR and there are no data on complete microbiological profiles of patients enrolled.

## 4. Conclusions

This article reports firstly a correlation between MetS presence and a greater prevalence of some bacterial species in the peri-implant sulcus, irrespectively from peri-implant status (health vs. disease).

Understanding the existing relationship between peri-implant diseases and metabolic syndrome could lead to the implementation of preventive measures and a more conscious approach while treating these patients. Further studies with a longitudinal design, to make up for the spatiotemporal changes in bacterial profile, larger sample and analyzing more microbial species are required to properly highlight the relationship suggested by our findings.

## 5. Materials and Methods

### 5.1. Study Design

To address the research purpose, the authors developed and implemented a case-control study, conducted at “Sapienza” University of Rome.

### 5.2. Study Population

From April 2019 to September 2019, all subjects referred to “Sapienza” University of Rome for screening, diagnosis, and treatment of MetS were consecutively evaluated by an experienced physician (CL).

Patients were enrolled in the study based on the following inclusion criteria: age ≥ 18 years, presence of at least one osseointegrated implant with >5 years functional loading. Patients were excluded if they had an implant with less than 5 years of functional loading or refused to be subjected to a dental examination at the Oral Surgery Unit.

A total of 229 consecutive patients were referred at the Oral Surgery Unit in order to evaluate peri-implant status: 131 did not meet the inclusion criteria, 26 refused to be included in the study, 22 did not attend the scheduled visit and refused a new dental examination.

Therefore, a final sample of 50 patients was enrolled in the study: thirty-eight females and fourteen males, with a mean age of 57.17 ± 8.44 years (range: 45–71 years). In the medical history 25 patients had a diagnosis of metabolic syndrome, while the remaining 25 did not meet MetS criteria. Detailed demographics of sample enrolled are reported in Table 4.

Each patient received detailed descriptions of the study protocol and all subjects signed the inform consent form and gave written approval to be included in the study population, according to the latest version of the World Medical Declaration of Helsinki (2013). The study was approved by the institution review board of “Sapienza” University of Rome (Ref. 4948/2018) and reported according to the STROBE statement (www.strobe-statement.org (accessed on 14 January 2021)).

### 5.3. Medical Examination

Anthropometric measurements and venous blood samples were obtained from all patients in the early morning after an overnight fast. Experienced physicians (CL, CL) blinded with respect to periodontal and peri-implant conditions performed the anthropometric measurements. Body Mass Index (BMI) was recorded for each patient (Kg/m^2^): body height was recorded to the nearest 0.5 cm and body weight to the nearest 0.1 kg.

Waist circumference was measured placing the measuring tape horizontally around the patient’s abdomen and aligning the bottom edge of the tape with the belly bottom and rounded to the nearest 0.1 cm. A measuring tape with a spring handle was used in order to control the pressure exerted on the patient’s abdomen. The same calibrated investigator expert in the field (CL) repeated all measurements, the calibration was accepted when repeated measurements (*n* = 10) presented an intraclass correlation coefficient (ICC) greater than 0.85. Data about smoking habit, as well as current medications (number and type), past medical history, was collected by trained staff.

Serum concentrations of fasting plasma glucose, total cholesterol, high density lipoprotein (HDL) cholesterol, low density lipoprotein (LDL) cholesterol and triglycerides (TG) were measured collecting up to 5 mL of blood samples for each patient.

The 24-h ABPM was performed using the Spacelabs 90207 (SpaceLabs^®^, Snoqualmie, WA, USA): for each registration, blood pressure (BP) values were obtained every 15 min during the day and every 30 min during the night time period.

All patients were screened for MetS according the NCEP ATP III criteria [36]. The diagnosis was made by the evidence of ≥3 of the following criteria: (1) WC ≥ 102 cm (M) or ≥ 88 cm (F); (2) Fasting plasma glucose value ≥ 110 mg/dL; (3) serum triglycerides concentration ≥ 150 mg/dL; (4) serum HDL-cholesterol concentration < 40 mg/dL (M) or <50 mg/dL (F) and (5) BP ≥ 130/85 mmHg, obtained by 24-h Ambulatory blood pressure monitoring.

### 5.4. Periodontal and Peri-implant Clinical Examination

A full mouth periodontal examination was conducted for all patients enrolled. For each implant, the following clinical measurements were also recorded at six sites per implant using a periodontal probe (PCP-Unc 15, Hu-Friedy^®^, Chicago, IL, USA) with a light force (approximately 0.15 N) by the same trained calibrated operator (BDM):

Probing Pocket Depth (PPD) Measured in millimeters;

Plaque Index (PI) recorded with dichotomic values (present/absent);

Mucosal redness (MR) recorded with dichotomic values (present/absent);

Suppuration recorded with dichotomic values (present/absent);

Bleeding on probing (BOP) recorded with dichotomic values (present/absent).

To achieve intra-examiner reliability, the examiner was calibrated to show an agreement of 90% within 1 mm by duplicate measurements of probing depths on randomly selected teeth (10) and implants (10).

Case definition of periodontitis and peri-implant diseases were confirmed at the visit using the 2017 World Workshop on the Classification of Periodontal and Peri-Implant Diseases and Conditions criteria for epidemiological studies [5,37]. Furthermore, years of functional loading, implant location (maxilla or mandible) and type of prostheses (single crown or multiple unit) were recorded.

### 5.5. Radiographic Evaluation

In addition, mesial and distal implant crestal bone levels were measured on digital periapical x-rays for each implant obtained by using an imaging plate scanner (PSPIX²^®^, Acteon Group, Norwich, UK). A calibrated software (SOPRO Imaging, Acteon Group, Norwich, UK) was used to estimate marginal bone level. Two expert investigators who were blinded to other aspects of the study conducted the radiographic assessment. Any disagreement was solved by consensus, and a third investigator was consulted when it was not initially possible to achieve complete agreement (defined as a difference between the measurements made by the two experts of >0.1 mm). The reference point for the bone level measurement was the implant shoulder. The bone level was digitally evaluated by measuring the distance between the implant shoulder and the first visible bone contact on the implant at the mesial and distal aspect of each implant.

### 5.6. Microbiological Sampling and Analysis

For each patient, microbiological samples were collected from the deepest pockets of two implants: one healthy and one affected by peri-implant diseases (mucositis and peri-implantitis).

If a patient had no healthy implants, then the samples were taken from a healthy tooth of the same quadrant (pockets < 4 mm, no bleeding on probing).

Peri-implant sulcular biofilm samples were obtained by inserting two sterile endodontic ISO #40 paper points (Roeko; Langenau, Germany) in the deepest aspect of the peri- implant sulcus for 30 s. They were, then, stored on ice in a sterile 2.0 mL Eppendorf transport tube (Eppendorf AG; Hamburg, Germany), and promptly delivered to the Department of Experimental Medicine. The quantitative real-time polymerase chain reaction (PCR) was performed by two experienced researchers (MM, EDS) to evaluate total bacterial count of six pathogens: *A. actinomycetemcomitans (Aa)*, *P. intermedia (Pi)*, *P. gingivalis (Pg)*, *F. nucleatum (Fn)*, *Treponema denticola (Td)* and *Staphylococcus aureus (Sa).*

Bacterial DNA was, then, extracted from each dental swab (*n* = 100) using ISOLATE II Genomic DNA Kit (Bioline, London, UK) according to the manufacturer’s instructions.

To establish standard curves for quantification by real-time PCR, each of the six bacterial DNAs, purchased from ATCC (ATCC, VA, USA) were diluted in eight two-fold dilutions. The clinical DNA samples together with the serial five-fold dilutions of genomic DNA from the six target species, were analyzed in triplicate in 96well plates using a ViiA7^TM^ Real-Time PCR system (Thermo Fisher Scientific, Waltham, MA, USA). The mixtures contained 2 uL of template DNA, 12.5 uL of SensiFAST SYBER Hi-ROX Kit (Bioline, London, UK); 0.5 uL of each primer pair (10 uM) and 10 uL of ddH_2_O (Vf 20 uL). The amplification cycling conditions used were 95 °C for 3 min, then 40 cycles of 95 °C for 5 s, 62 °C 30 s. The primers’ information is listed in Table 5. The specificity of the PCR products was verified by analyzing the respective melting temperature profiles. Results were calculated using the ViiA7^TM^ SoftWar (Thermo Fisher Scientific, Waltham, MA, USA) based on the standard curves constructed for each of the six target species.

### 5.7. Statistical Analysis

Data were evaluated using standard statistical analysis software (version 20.0, Statistical Package for the Social Sciences, IBM Corporation, Armonk, NY, USA). A database was created using Excel (Microsoft, Redmond, WA, USA). Descriptive statistics including mean ± SD values and percentage were calculated for each variable. The Shapiro–Wilk test [38] was used to determine whether or not the data conformed to a normal distribution. The Mann–Whitney test [39] was used to evaluate inter-group differences between clinical, microbiological and radiographic parameters. In each test, the cut-off for statistical significance was *p* ≤ 0.05, with a beta power of 0.90.

## Figures and Tables

**Table 1:** Implant characteristics of patients enrolled.

Variable	*n*
Follow-up (years)	6.47 ± 4.56
Probing pocket depth (mm)	3.23 ± 1.15
Marginal bone loss (mm)	1.27 ± 1.12
Plaque Index (%)	37.82
Bleeding on Probing (%)	54.48
Mucosal Redness (%)	33.97
Suppuration (%)	10.89
Type of prosthesis	
Single crown (n)	92
Multiple unit (n)	64
Implant location	
Maxilla (n)	89
Mandible (n)	67
Peri-implant status	
Peri-implant mucositis (%)	42
Peri-implantitis (%)	26
Healthy implants (%)	32

**Table 2:** Total bacterial counts dividing patients in groups by peri-implant status (healthy vs. peri-implant disease).

	Healthy	Peri-Implant Disease	*p* Value
*Aa*	4.2	8.5	>0.05
*Pi*	1179.8	6484.7	0.0332
*Pg*	47579.4	91757	<0.001
*Sa*	16.8	25.4	>0.05
*Td*	31125.5	24765.5	<0.001
*Fn*	11488.1	47788.4	<0.001

Aa: *Aggregatibacter actinomycetemcomitans*; Pi: *Prevotella intermedia*; Pg: *Porphyromonas gingivalis*; Sa: *Staphylococcus aureus*; Td: *Treponema denticola*; Fn: *Fusobacterium nucleatum*.

**Table 3:** Total bacterial counts dividing patients in groups by metabolic status (Metabolic syndrome vs. healthy).

	Healthy	Metabolic Syndrome	*p* Value
*Aa*	4.2	8.5	0.0008
*Pi*	166	6074.2	0.0477
*Pg*	16631.7	82340.1	>0.05
*Sa*	14.2	27.1	0.034
*Td*	17260.1	37231.7	>0.05
*Fn*	13994.5	41479.1	>0.05

Aa: *Aggregatibacter actinomycetemcomitans*; Pi: *Prevotella intermedia*; Pg: *Porphyromonas gingivalis*; Sa: *Staphylococcus aureus*; Td: *Treponema denticola*; Fn: *Fusobacterium nucleatum*.

**Table 4:** Sample demographics.

Variable	*n*	%
*Gender*		
Male	13	26
Female	37	74
*Diagnosis of metabolic syndrome*		
Yes	25	50
No	25	50
*Presence of periodontitis*		
Yes	32	64
No	18	36
*Smoking*		
Yes	10	20
No	40	80

**Table 5:** Primers’ information for the real-time PCR.

Primer	Strain ATCC n°	Sequence (5′-3′)	Tm
*Td*	33520	AGAGCAAGCTCTCCCTTACCGT TAAGGGCGGCTTGAAATAATGA	60
*Pg*	33277	TACCCATCGTCGCCTTGGT CGGACTAAAACCGCATACACTTTG	65
*Aa*	29523	CTTACCTACTCTTGACATCCGAA ATGCAGCACCTGTCTCAAAGC	65
*Fn*	25586	CGCAGAAGGTGAAAGTCCTGTAT TGGTCCTCACTGATTCACACAGA	65
*Pi*	25611	CGTGGACCAAAGATTCATCGGTGGA CCGCTTTACTCCCCAACAAA	60
*Sa*	700698	GCGATTGATGGTGATACGGTT AGCCAAGCCTTGACGAACTAAAGC	65

Aa: *Aggregatibacter actinomycetemcomitans*; Pi: *Prevotella intermedia*; Pg: *Porphyromonas gingivalis*; Sa: *Staphylococcus aureus*; Td: *Treponema denticola*; Fn: *Fusobacterium nucleatum*; Tm: *melting temperature*.

## Data Availability

The data presented in this study are available on reasonable request from the corresponding author.
